# Isolation of RNA from equine peripheral blood cells: comparison of methods

**DOI:** 10.1186/2193-1801-2-478

**Published:** 2013-09-22

**Authors:** Zibin Jiang, Cornelius E Uboh, Jinwen Chen, Lawrence R Soma

**Affiliations:** Department of Clinical Studies, University of Pennsylvania School of Veterinary Medicine, New Bolton Center Campus, 382 West Street Road, Kennett Square, PA 19348 USA; Pennsylvania Equine Toxicology & Research Center, Department of Chemistry, West Chester University, West Chester, PA 19382 USA

**Keywords:** RNA extraction, Equine, Blood, LeukoLOCK™, Method comparison

## Abstract

Gene expression studies in equine research involve the use of whole blood samples as a vital source of RNA. To determine the optimal method for RNA isolation from equine whole blood, we compared three RNA isolation strategies using different commercially available kits to evaluate the yield and quality of equine RNA. All 3 methods produced RNA with high quality. Though it did not produce the highest yield, combining the quality, yield and the need for the downstream application in our project, LeukoLOCK™ total RNA isolation system was the best RNA extraction method.

**Electronic supplementary material:**

The online version of this article (doi:10.1186/2193-1801-2-478) contains supplementary material, which is available to authorized users.

## Introduction

RNA is a particularly labile macromolecule prone to degradation, and RNA degradation can result in falsely altered gene expression patterns (Catts et al. 2005). For this reason, extraction of cellular RNA is a critical step in the search for molecular information pertinent to gene expression and biomarker research.

For our studies, whole blood is the most practical source of RNA. However, it is a challenging medium since pertinent RNA information primarily resides in the small number of circulating white blood cells (WBC), which comprise only a small fraction of whole blood cellular fraction.

Currently, there are commercially available kits that are used to isolate and purify RNA. However, there are few studies available to date that have compared RNA extraction methods from samples of animal origin. Extraction methods from swine tissue (Deng et al. 2005) and bovine blood (Hammerle-Fickinger et al. 2010) have been compared, but, there are no peer-reviewed reports on comparing methods for isolating RNA from equine blood. In this report, 3 commercially available kits based on different isolation strategies were compared with respect to the yield and quality of RNA. The quality of the RNA isolate was assessed for purity and intactness. Furthermore, the presence of trace DNA contamination was determined using real-time PCR.

## Methods

### Ethics statement

The study protocol was approved by the University of Pennsylvania Institutional Animal Care and Use Committee.

### Method 1: RNA extraction using LeukoLOCK™ total RNA isolation system

Ten mL fresh blood was collected in a heparinized tube and RNA was extracted using LeukoLOCK***™*** total RNA isolation system (ABSciex Inc.) following the manufacturer’s instructions (for details see Additional file 1).

### Method 2: RNA extraction from equine whole blood using Tempus™ blood RNA tube

Three mL fresh equine blood was collected in a Tempus***™*** blood RNA tube and vigorously shaken for 30 sec. The lysate was used to extract RNA using ABI 6100 Nucleic Acid PrepStation (ABSciex Inc.) following the manufacturer’s instructions (for details see Additional file 1).

### Method 3: RNA extraction from equine whole blood using TRIzol® reagent

Ten mL of equine blood was collected into a heparinized tube, centrifuged at 2000 × g at 4°C for 10 min. Plasma was removed and WBC were transferred into a 15 mL tube. 3-times the volume of RBC lysis buffer (Qiagen) was added. The tube was vortexed for 15 sec, and incubated at room temperature for 15 min prior to centrifugation at 2000 × g for 10 min (4°C). Supernatant was discarded, the isolation of RNA from WBC pellet was performed using TRIzol® reagent (Invitrogen) following the manufacturer’s instructions (for details see Additional file 1).

### Determination of RNA concentration and Evaluation of RNA purity using SmartSpec*™* 3000 Spectrophotometer (Bio-RAD)

Two μL of RNA was diluted 1:200 with TE buffer. The RNA assay mode was selected in SmartSpec***™*** 3000 Spectrophotometer; the conversion factor was set as 1.0 at an absorbance of 37.0 μg/mL. The TE buffer was used as blank and the RNA concentration was determined at 260 nm and 280 nm with a ratio of A_260nm_/A_280nm_.

### Evaluation of RNA integrity using Agilent 2100 Bioanalyzer (Agilent)

Three μL of RNA was denatured at 65°C for 2 min and 1 μL of denatured RNA and 5 μL RNA 6000 Nano markers were added to Agilent RNA 6000 Nano chip. The chip was vortexed for 1 min and loaded to Agilent 2100 Bioanalyzer. RIN and peaks of 18 S and 28 S for each RNA isolate were checked using the Agilent 2100 Nana RNA assay software.

### Test for DNA contamination and RNA quality using agarose RNA denaturing gel

RNA (5 μL) was mixed with 1 μL 6x gel-loading buffer and electrophoresed on 1.2% agarose gel in 1x MOPS running buffer (pH 7.0). Electrophoresis was performed at 100 V for 1.0 hour using Bio-Rad Power Pac 200 power source (Bio-Rad). Gel was stained with ethidium bromide. Images of the developed gels were acquired by Foto/Analyst luminary 12-bit cooled Camera Electronic Documentation System (Fotodyne Incorporated).

### Detection of traces of DNA contamination using real-time PCR

Aliquots of total RNA were amplified using specific primers corresponding to regions of equine IL-6 and internal control GAPDH. The sequence of the forward primer for IL-6 was: AACAACTCACCTCATCCTCGTAA, whereas that of the reverse primer for IL-6 was CGAACAGCTCTCAGGCTGAAC. The sequence of the forward primer for GAPDH was CAAGGCTGTGGGCAAGGT, whereas that of the reverse primer for GAPDH was GGAAGGCCATGCCAGTGA. The reverse transcription was carried out in 20 μL reaction mixtures, including 10 μL RNA, 2.0 μL reverse transcription buffer, 0.8 μL 25x dNTPs, 2.0 μL 10x random primers, and 50 U MultiScribe Reverse Transcriptase**.** Each reaction mixture was incubated at 37°C for 2 hr, then at 85°C for 5 seconds. The PCR step was carried out in 25 μL, including 25 μL 2x TaqMan® Universal PCR Master Mix, 50 nM forward primer, reverse primer and TaqMan® probe, and 5 μL cDNA sample. The reaction mixtures were heated at 95°C for 10 min and then subjected to 40 cycles at 95°C for 15 sec, and 60°C for 1 min. The reaction mixture was loaded onto 7500 Real-Time PCR System (ABSciex Inc.). Method of relative quantitation of gene expression was adopted, and RNA isolate extracted with LeukoLOCK***™*** total RNA isolation system was used as reference.

## Results

Mean yield and ratio of absorbance (A) _260nm_/A_280nm_ of RNA are shown in Table 1. The TRIzol® reagent method produced the highest RNA yield (3.78 μg per mL of whole blood), while the other two methods LeukoLOCK***™*** total RNA isolating system and Tempus***™*** blood RNA tube produced lower but equivalent, 0.99 and 1.09 ug per ml of whole blood, respectively. The ratio of A_260nm_/A_280nm_ for all 3 methods was > 1.8, indicating that there was no contamination of RNA due to protein and other organic molecules.

**Table 1:** **Comparison of the average (mean ± SD) yield and ratio of A**_**260nm**_ **/A**_**280nm**_ **of RNA Isolate using 3 different methods; LeukoLOCK™, Tempus™, and TRIzol® reagent**

Methods	LeukoLOCK***™***(n=10)	Tempus***™***(n=10)	TRIzol® reagent (n=10)
**A**_**260nm**_ **/A**_**280nm**_	**2.06 ± 0.05**	**1.97 ± 0.06**	**1.98 ± 0.06**
**Yield (μg/ml)**	**0.99 ± 0.07**	**1.08 ± 0.09**	**3.78 ± 0.12**

The ratios of A_260nm_/A_280nm_ for the 3 RNA extracts from the 3 different methods were > 1.80, indicating high RNA purity without contamination by protein and other organic molecules.

**Figure 1:**
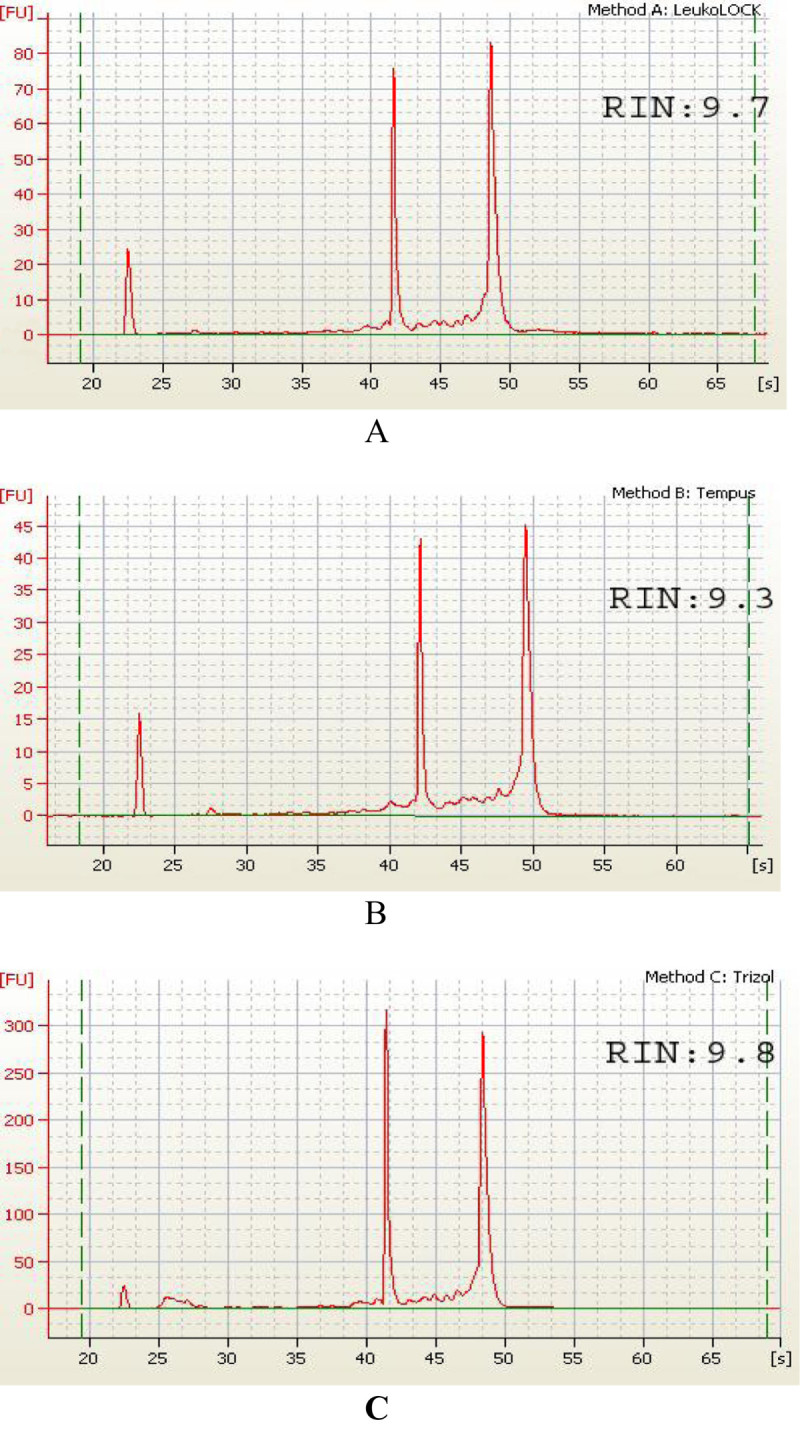
**Electropherograms of RNA. A**: RNA extract using Method 1 (LeukoLOCK™ total RNA isolation system); **B**: RNA extract using Method 2 (Tempus™ blood RNA tube with ABI PRISM® 6100 Nucleic Acid PrepStation); **C**: RNA extract using Method 3 (TRIzol® reagent). In evaluating RNA using Agilent 2100 Bioanalyzer, RNA with high quality showed distinct 18S and 28S peaks, and RIN > 8.0. In the present study, all samples showed distinct 18 S and 28 S peaks, and RINs were 9.7, 9.3, and 9.8, respectively, indicating that the RNA isolates had high integrity with negligible RNA degradation.

**Figure 2:**
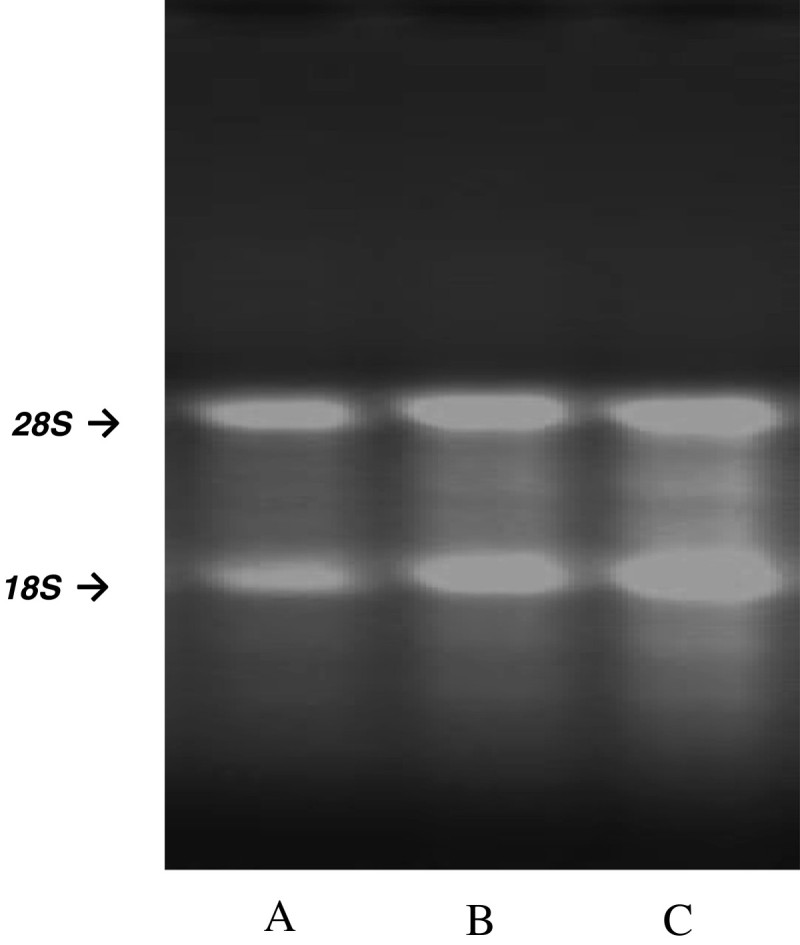
**Total RNA quality assessment using agarose denaturing gel electrophoresis. A**: RNA extract using Method 1 (LeukoLOCK*™* total RNA isolation system); **B**: RNA extract using Method 2 (Tempus*™* blood RNA tube with ABI PRISM® 6100 Nucleic Acid PrepStation); **C**: RNA extract using Method 3 (TRIzol® reagent). RNA extracts from all 3 methods (1, 2 and 3) showed distinct 18 S and 28 S bands without DNA contamination specified by DNA bands.

Electropherograms of RNA isolates obtained from the 3 different methods are shown in Figure 1. It should be noted that the isolated RNA from all samples exhibited distinct 18S and 28S peaks, with RNA integrity numbers (RIN) >8, indicating that the integrity of all the RNA extracts was acceptable. Furthermore, 3 different RNA extracts with 1.2% denaturing agarose electrophoresis were evaluated. Presence of distinct 18 S and 28 S bands without DNA bands, suggested the absence of DNA contamination (Figure 2).

**Figure 3:**
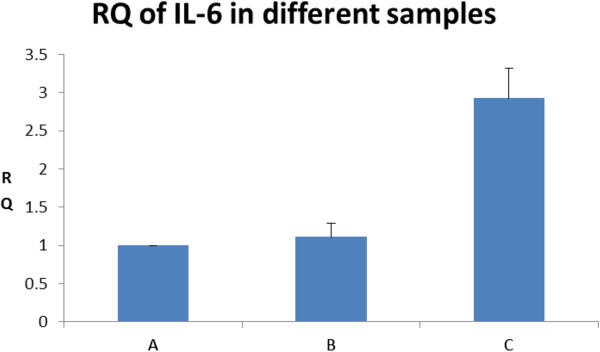
**Relative expression of IL-6 in the RNA extracts from 3 different isolation methods (n=5). A**: RNA extract using Method 1 (LeukoLOCK*™* total RNA isolation system); **B**: RNA extract using Method 2 (Tempus*™* blood RNA tube with ABI PRISM® 6100 Nucleic Acid PrepStation); **C**: RNA extract using Method 3 (TRIzol® reagent). Sample A was control. All the samples were extracted from the same host at the same time. The relative expression level of IL-6 in Sample **B** was 1.11±0.18, and that of **C** was 2.93±0.39, suggesting that there was a trace of DNA contamination in sample **C**, but not in **B**.

**Figure 4:**
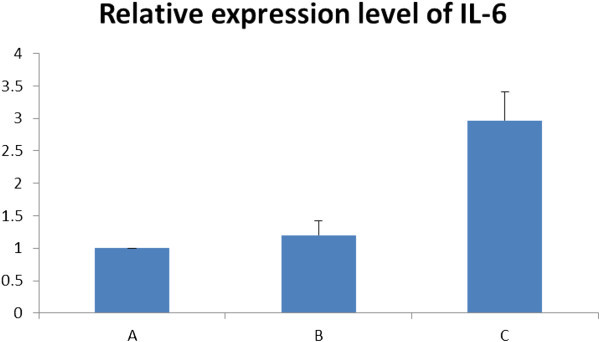
**Relative expression levels of IL-6 after Dnase treatment (n=5). A**: RNA Samples extracted using Method 1 (LeukoLOCK*™* total RNA isolation system); **B**: RNA samples extracted using Method 3 (TRIzol® reagent) and then treated with Dnase; **C**: RNA samples extracted using Method 3 (TRIzol® reagent). Sample A was control. After sample **C** was treated with Dnase, the relative expression level of IL-6 (sample B) was reduced to 1.20 ± 0.22, indicating that DNA contamination in sample **C** was eliminated.

Contamination by DNA was not detected during evaluation but, considering the sensitivity and limitation of these methods, real-time PCR was performed in order to determine if trace DNA contamination was present or not. For this purpose, Interleukin (IL)-6 was used as the target gene, and housekeeping gene, GAPDH, was used as endogenous control. The pair of primers for IL-6 was designed based on Exon 5. Expression of IL-6 in the RNA extract using TRIzol® reagent was nearly 3-times higher than the RNA isolates obtained from the other 2 methods (Figure 3). All the samples were extracted from the same host at the same time. Real-time PCR results obtained suggested that there was trace DNA contamination in the RNA extract obtained using TRIzol® reagent. Following Dnase treatment, the relative expression of IL-6 in TRIzol® reagent RNA extract was reduced further confirming that DNA contamination was present in TRIzol® reagent-derived RNA (Figure 4).

## Discussion

Various methods and kits are available for the extraction of RNA from cells, blood, tissue, and plasma. In the present study, 3 different commercially available kits for the isolation of RNA from equine whole blood based on different strategies were compared. For Tempus*™* blood RNA tube with RNA preparation station, whole blood was directly lysed in Tempus*™* blood RNA tube containing lysis buffer, and RNA was extracted from lysed whole blood solution. For the other two methods, WBC were separated from plasma and red RBC before extracting RNA.

High quality RNA is critical for downstream application, and the quality depends upon the technique that was employed for RNA isolation (Fleige and Pfaffl 2006). Quality of total RNA was assessed on the basis of RNA intactness and purity; 4 methods were used for assessment: (1) spectrophotometry, (2) micro-capillary chip's electrophoresis with fluorescent detection (Bioanalyzer, Agilent Technologies), (3) RNA denaturing gel electrophoresis and (4) real-time PCR.

The intactness of RNA extract was assessed by analyzing 18S and 28S subunits of ribosomal RNA on micro-capillary chip electrophoresis with fluorescent detection and on ethidium stained agarose denaturing gel electrophoresis (Strand et al. 2007 Schroeder et al. 2006 and Dumur et al. 2004), while purity of RNA was determined by calculating the ratio of A_260nm_/A_280nm_ using spectrophotometer (Glasel 1995). Purity of RNA was considered adequate if the ratio was > 1.8 (Manchester 1996). In most cases, RNA samples assessed by these 3 methods would be recommended for use in downstream molecular analysis. In the present comparative study, the 3 methods above were not adequate for the assessment of RNA quality. Although, there was no DNA contamination using RNA agarose denaturing gel (Figure 2), considering the limit and sensitivity of agarose denaturing gel and the absence of Dnase treatment of the RNA extracts using TRIzol® reagent, a forth method, the relative IL-6 expression level in different RNA extracts was compared using real-time PCR.

The primer pair for IL-6 was from the same exon. If there were DNA contamination, the apparent IL-6 expression level would be falsely higher than the actual level. Real-time PCR results suggested that there was DNA contamination in the RNA extracts using the TRIzol® reagent method (Figure 3). Treating contaminated RNA extract with Dnase, the expression level of IL-6 was reduced. This observation further confirmed that trace DNA contamination occurred in the RNA isolate when the TRIzol® reagent method was used.

Some downstream applications do not tolerate any trace of DNA contamination, therefore the RNA isolates should be assessed by real-time PCR to determine whether or not trace DNA contamination has occurred, which is an index for demonstrating the quality and purity of the RNA extraction.

Comparing the RNA yield from the 3 methods, TRIzol® reagent method produced the highest, whereas the yield from the other two methods were equivalent but lower. For the TRIzol® reagent method WBC were separated from whole blood and almost all the WBC were recovered. For Tempus*™* method, whole blood was lysed in their blood RNA tubes. The lysate contained high concentrations of components, and another cycle of RNA precipitation and dissolution was performed. All these factors will contribute to the reduction in RNA yield. For LeukoLOCK*™* total RNA isolation method, WBC were separated from whole blood by filtration. The waste tubes containing plasma and RBC were centrifuged at 2000 × g for 10 min, and WBC layer was observed in the waste tube. The WBC in the waste tubes indicated that not all WBC could be captured by the filter. The present procedure resulted in decreased RNA yield.

High concentrations of globin transcripts in blood can confound the accurate assessment of the expression levels of genes harvested from blood (Wright et al. 2008). Abundant globin mRNA represents up to 70% of the total expressed transcripts and consequently limits the accurate detection of genes expressed at low concentrations. Thus, globin reduction is often considered a necessary step in the evaluation of whole-blood gene expression profiles via microarray assay. It has been shown that reduction of abundant globin mRNA is essential for revealing unique patterns of gene expression (Winn et al. 2010). Globin mRNA exists in reticulocytes (Guesella et al. 1979). LeukoLOCK*™* total RNA isolation method removes plasma, RBC and reticulocytes, hence RNA recovered contains only minor quantity of globin mRNA. For this reason, when used in microarray assay, globin mRNA depletion procedure is not necessary. For RNA samples extracted using the other two methods, the globin mRNA depletion must be conducted before microarray assay is performed.

In this study, gene expression profiling with microarray was evaluated and validated using real-time PCR. Comparing the features of the three methods described, LeukoLOCK*™* total RNA isolation system method was the best choice.

## Electronic supplementary material

Additional file 1: Protocols for RNA extraction. (DOCX 22 KB)

Below are the links to the authors’ original submitted files for images.

Authors’ original file for figure 1

Authors’ original file for figure 2

Authors’ original file for figure 3

Authors’ original file for figure 4

## Abbreviations

A: Absorbance
GAPDH: Glyceraldehyde 3-phosphate dehydrogenase
DNA: Deoxyribonucleic acid
Dnase: Deoxyribonuclease
IL: Interkeukin
mRNA: Messenger Ribonucleic acid
MOPS: 3-(N-morpholino) propanesulfonic acid
PCR: Polymerase chain reaction
RBC: Red blood cells
RIN: Ribonucleic acid integrity number
RNA: Ribonucleic acid
RQ: Relative quantitation
WBC: White blood cells.
