# Supplementary material for: Isolation of RNA from equine peripheral blood cells: comparison of methods
Source: Springerplus. 2013 Sep 22;2(1):478. doi: 10.1186/2193-1801-2-478 (PMC3797321; doi:10.1186/2193-1801-2-478)
Supplement: Supplementary file 1 — Additional file 1: Protocols for RNA extraction. (DOCX 22 KB) [file 40064_2013_574_MOESM1_ESM.docx]

**Isolation of RNA from equine peripheral blood cells: Comparison of methods**

**Zibin Jiang^1^**^§^**, Cornelius E. Uboh^1,2^, Jinwen Chen^1^, and Lawrence R Soma^1^.**

1 University of Pennsylvania School of Veterinary Medicine, Dept. of Clinical Studies, New Bolton Center Campus, 382 West Street Road, Kennett Square, PA 19348, USA

2 Pennsylvania Equine Toxicology & Research Center, Dept. of Chemistry, West Chester University, West Chester, PA 19382, USA

^§^Corresponding author:

Dr. Lawrence R Soma

Tel 610-925-6265; Fax 610-925-6820

E-mail: soma@vet.upenn.edu

Journal: Biotechnology Letters

Journal section: Animal Cell Technology

# Methods

**Method 1: RNA extraction using LeukoLOCK^TM^ total RNA isolation system**

Ten mL fresh blood was collected in a heparinized tube and RNA extracted using LeukoLOCK^TM^ total RNA isolation system [25]. Briefly, whole blood was passed through LeukoLOCK^TM^ filter, the filter flushed with 3 mL phosphate buffered saline (PBS) and 3 mL of RNAlater^®^. The residual RNAlater^®^ was removed from the LeukoLOCK^TM^ filter and the filter flushed with 2.5 mL pH-adjusted Lysis/Binding Solution. The lysate was collected into 15 mL tubes containing 2.5 mL nuclease-free water; 25 μL proteinase K was added and shaken for 5 min on a roto-rack. RNA binding beads (50 μL) and 2.5 mL 100% isopropanol alcohol were then added, incubated at room temperature for 5 min, and centrifuged at 2000 x g for 3 min. Supernatant was discarded, RNA binding beads washed with 1.2 mL washing solution and transferred to a 1.5 mL Eppendorf tube. The tube containing the binding beads was centrifuged at 16000 x g for 15 seconds, the supernatant discarded, RNA binding beads washed with 750 μL of the 2/3 washing buffer, and air dried. Dnase mixture (300 μL) was added to the tube and incubated for 10 min at room temperature; 300 μL lysis buffer solution and 300 μL 100% isopropanol alcohol were added and re-incubated for 3 min at room temperature, centrifuged at 16000 x g for 15 seconds, and the supernatant discarded. RNA binding beads were washed 2-times with 750 μL of the 2/3 washing solution and air dried for 3 min. RNA was recovered from RNA binding beads with 80 μL by elution and stored at –80°C.

**Method 2: RNA extraction from equine whole blood using Tempus^TM^ blood RNA tube**

Three mL fresh equine blood was collected in a Tempus^TM^ blood RNA tube and vigorously shaken for 30 seconds. The lysate was poured into a 50 mL Rnase-free tube [26], 3 mL 1X PBS added, and vortexed vigorously for 30 seconds. RNA was subsequently isolated using ABI 6100 Nucleic Acid PrepStation. Briefly, the lysate diluted with 1X PBS was passed through the 20 mL reservoir of the filter. The filter was washed with 3 mL RNA purification wash solution-1 and 4.5 mL RNA purification wash solution-2. Washing with washing solution-1was repeated until the filter membrane was free of red or brown heme pigmentation. Absolute RNA (0.35 mL) wash solution was added to the filter membrane and incubated for 15 min at room temperature. The filter was washed 3-times with 3 mL RNA purification wash solution, vacuum dried, and RNA eluted from the filter using 0.5 mL nucleic acid purification elution solution. RNA was precipitated with 100 % isopropanol alcohol from the 300 μL RNA elution, and recovered in 50 μL TE buffer (10 mM [Tris](http://en.wikipedia.org/wiki/Tris), 1 mM [EDTA](http://en.wikipedia.org/wiki/EDTA), adjust pH to 7.4 with [HCl](http://en.wikipedia.org/wiki/HCl)). The recovered RNA was stored at –80°C.

**Method 3: RNA extraction from equine whole blood using TRIzol^®^ reagent**

Ten mL of equine blood was collected into a heparinized tube, centrifuged at 2000 x g at 4 °C for 10 min. Three distinguished layers were formed; upper layer of plasma, a second thin buffy coat layer, and a lower layer of red blood cells. The buffy coat comprises < 1% of the total volume of the blood sample with WBC and platelets. Plasma was aspirated, buffy coat was transferred into a fresh 15 mL tube, and 3-times the volume of RBC lysis buffer added. The tube was votexed for 15 sec, and incubated at room temperature for 15 min prior to centrifugation at 2000 x g for 10 min (4°C). Supernatant was discarded, 1 mL RBC lysis buffer added to the cell pellet, followed by incubation at room temperature for 10 min. After centrifugation at 2000 x g for 10 min, at 4 °C, supernatant was discarded, WBC pellet lysed and homogenized with 1.0 mL TRIzol^®^ reagent [27]. The lysate was transferred into a 1.5 mL Eppendorf tube and incubated at room temperature for 5 min. Chloroform (0.2 mL) was added to the lysate, shaken vigorously for 15 sec and incubated at room temperature for 5 min. The sample was centrifuged for 15 min. at 12,000 x g at 4 °C. The upper layer was transferred to 1.5 mL fresh tube, mixed with 0.5 mL 100% isopropanol alcohol, and incubated at room temperature for 10 min. The mixture was centrifuged for 10 min at 12000 x g at 4 °C. The white RNA pellet was visible on the side of the tube. The pellet was washed with 1 mL 75% ethanol and centrifuged at 7500 x g for 5 min. at 4 °C. The pellet was air dried briefly, and dissolved in 100 μL TE buffer. The recovered RNA was stored at –80°C.
