# Supplementary figures and images for: Isolation of RNA from equine peripheral blood cells: comparison of methods
Source: Springerplus. 2013 Sep 22;2(1):478. doi: 10.1186/2193-1801-2-478 (PMC3797321; doi:10.1186/2193-1801-2-478)

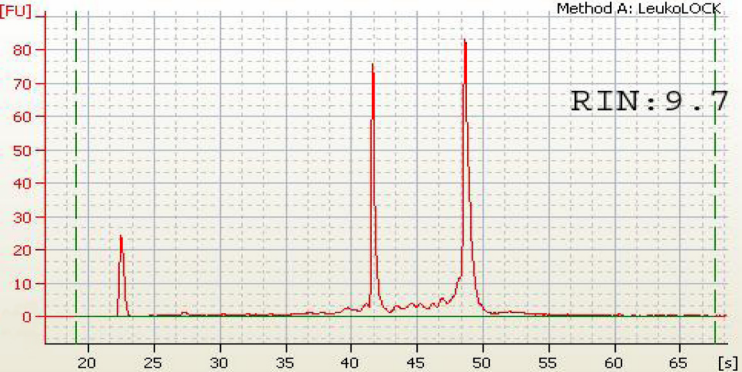

A

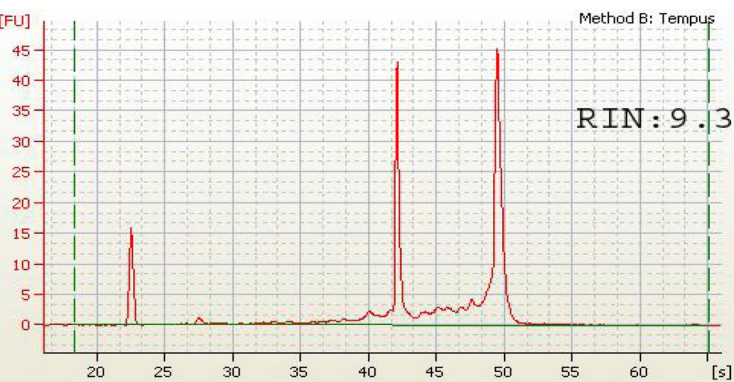

B

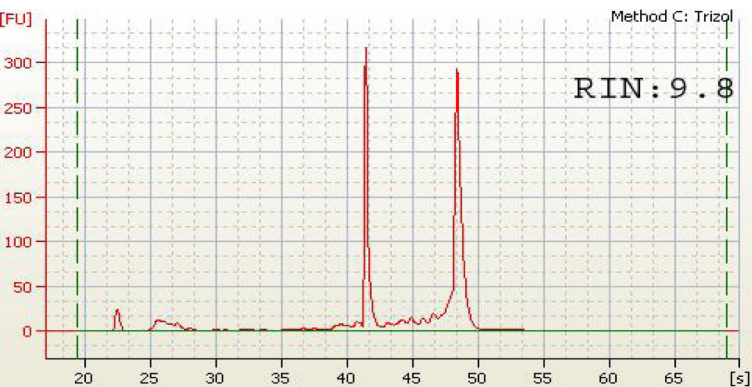

C

Supplement: Supplementary file 2 — Authors’ original file for figure 1 [file 40064_2013_574_MOESM2_ESM.pdf]

**28S →**

**18S →**

**A**

**B**

**C**

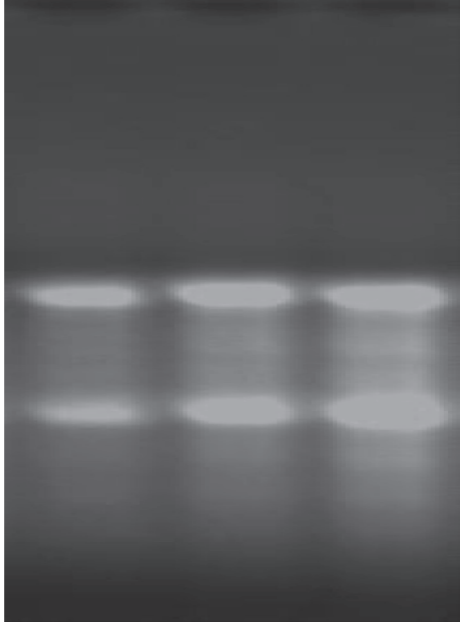

Supplement: Supplementary file 3 — Authors’ original file for figure 2 [file 40064_2013_574_MOESM3_ESM.pdf]

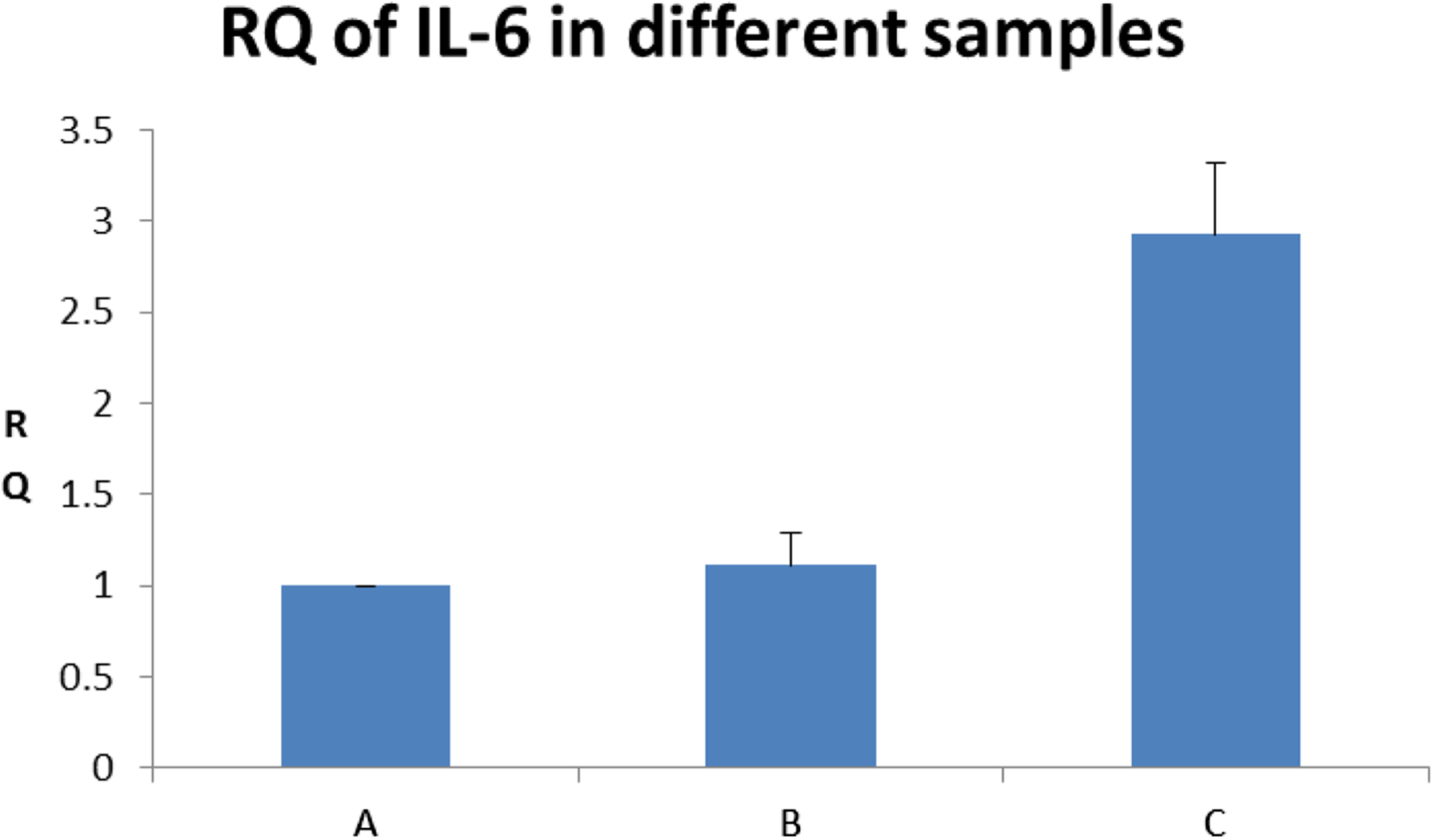

Supplement: Supplementary file 4 — Authors’ original file for figure 3 [file 40064_2013_574_MOESM4_ESM.tif]

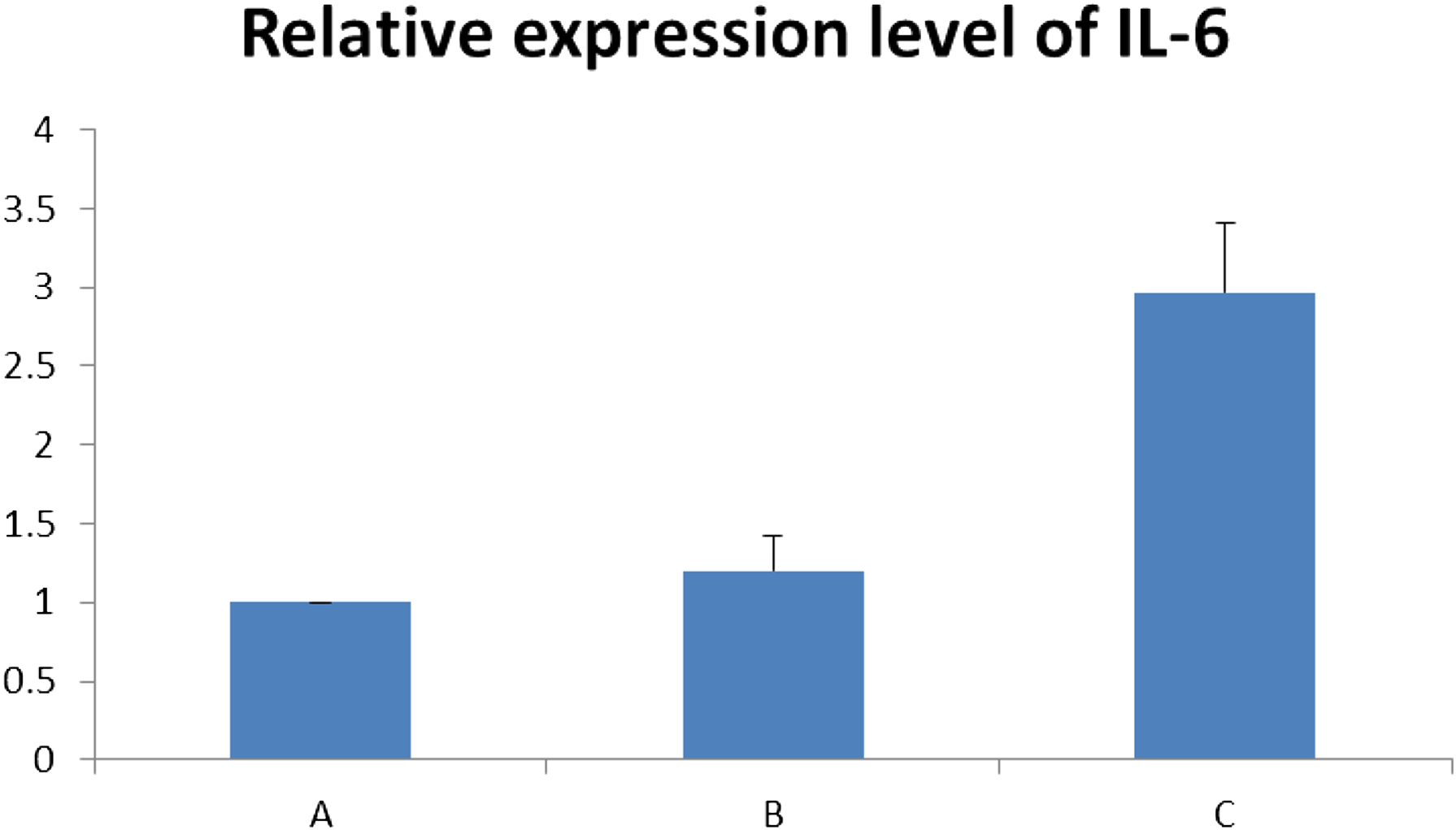

Supplement: Supplementary file 5 — Authors’ original file for figure 4 [file 40064_2013_574_MOESM5_ESM.tif]
